# Communication of Screen-Positive Newborn Bloodspot Screening Results for Metabolic Conditions in Ireland–Parent and Healthcare Professional Experience: A Service Evaluation

**DOI:** 10.3390/ijns12040078

**Published:** 2026-09-28

**Authors:** Mairéad Bracken-Scally, Anna O’Loughlin, Jenny McNulty, Olivia Walsh, Caoimhe Howard, Ahmad Ardeshir Monavari, Abigail Collins, Heather Burns

**Affiliations:** 1 National Healthy Childhood Programme, Health Service Executive (HSE), R35 TY28 Offaly, Ireland; 2 National Newborn Bloodspot Screening Laboratory, Children’s Health Ireland (CHI), D01 YC67 Dublin, Ireland; 3 National Centre for Inherited Metabolic Disorders, CHI at Temple Street, D01 XD99 Dublin, Ireland

**Keywords:** newborn screening, neonatal screening, dried blood spot screening, rare diseases, congenital metabolic disorder, parent experience, healthcare professional experience, clinician experience, communication of results, screen positive

## Abstract

Communication of screen-positive newborn bloodspot screening (NBS) results is inherently challenging for both parents and HCPs. There is no internationally accepted gold-standard model, with heterogeneity in practice and with many jurisdictions reporting suboptimal parent and HCP experience. This service evaluation aimed to assess the model for communication of initial screen positive results for metabolic conditions through the Irish National Newborn Bloodspot Screening Programme (NNBSP). Under the current model, screen-positive results are communicated to parents by a healthcare professional (HCP) (usually over the phone) and follow-up is arranged through the maternity hospital of birth. The evaluation required engagement with three cohorts, comprising two parent cohorts (a True Positive (TP) cohort and a False Positive (FP) cohort) and one HCP cohort. A separate survey was designed for each cohort, informed by a review of the international literature and subject matter expertise. Overall, our evaluation found that the current model for communication of screen-positive results is acceptable to HCPs, the majority of whom regard communicating these results as an appropriate part of their role (61%) and feel confident doing so (68%). However, many parents reported that they did not receive key information, and 70% wanted more information at this first communication. Parents wanted clear, reassuring, and actionable information, including details of the suspected condition, next steps, and where to seek further support. HCPs wanted additional resources to support communication of screen-positive results, including guidelines, expert contacts, condition-specific information resources, and checklists. The findings of this evaluation will inform revisions of the current model, aimed at ensuring a better experience for parents and HCPs.

## 1. Introduction

The Irish National Newborn Bloodspot Screening Programme (NNBSP) screens babies for rare conditions that benefit from early detection and intervention. Newborn bloodspot screening commenced in the Republic of Ireland in 1966, with the implementation of screening for phenylketonuria (PKU). At the time of this evaluation, the programme offered screening for nine rare conditions; PKU, homocystinuria (HCU), classical galactosaemia (CGal), maple syrup urine disease (MSUD), congenital hypothyroidism (CHT), cystic fibrosis (CF), glutaric aciduria type 1 (GA-1), medium chain acyl co-A dehydrogenase deficiency (MCADD), and adenosine deaminase deficiency severe combined immunodeficiency (ADA-SCID).

This service evaluation aimed to assess the model for communication of an initial screen-positive result for a metabolic condition to the parent/legal guardian through the Irish NNBSP. This evaluation was prompted by reports from clinical staff in the National Centre for Inherited Metabolic Disorders (NCIMD) of poor parent experience of initial communication of screen-positive results. Staff reported that parents of screen-detected babies with manageable metabolic conditions were attending initial appointments in significant distress, reporting confusion around the screening result, the condition suspected and implications for their baby. Some parents reported extreme dissatisfaction with the quality and quantity of information provided at initial communication of the screen-positive result, and some also reported that the method and manner of communication were also suboptimal.

Under the current model, screen-positive results for metabolic conditions are communicated to parents by a healthcare professional (HCP), and follow-up is arranged through the maternity hospital of birth (Ireland has a total of 19 public maternity hospitals/units) [1,2]. When a screen-positive result is reported in the National Newborn Bloodspot Screening laboratory (NNBSL), a clinical member of staff in the laboratory (the Clinical Liaison Nurse (CLN) or Director of the laboratory) contacts a designated liaison person (DLP), typically a nurse or pediatric registrar (a specialty trainee/resident physician), in the maternity unit/hospital where the baby was born by telephone and provides them with key demographic information for the baby and the name of the condition suspected. The DLP is asked to arrange for the parents to be contacted and informed of the screening result, what disorder is suspected, and why further testing is required. The parent may be contacted by the DLP themselves, or by another medical professional (doctor or nurse) in the maternity hospital; local practice varies. Parents are advised that their baby must be brought to hospital for review by the pediatric/neonatology team and are informed that their baby may require admission for a few days, depending on the results of the investigation. While the current model does not specify the method for contacting parents regarding screen-positive results, established practice is for the information to be communicated over the phone unless the baby is already being cared for as an in-patient.

Communication of positive NBS results is inherently challenging, as the diagnostic outcome is not yet known and screening programmes must seek to meet the information needs of parents whose children will ultimately receive a diagnosis (true positive screen) and those who will not (false positive screen). There is no gold standard model for communicating an initial screen-positive result to parents, with heterogeneity in practice between jurisdictions nationally and internationally and with many jurisdictions reporting suboptimal parent and HCP experience [3,4,5]. The, albeit limited, international literature demonstrates that receiving a screen-positive NBS result elicits discomfort [5], distress, anxiety, and trauma in parents [6,7]. While parental anxiety and concern is to be expected on receiving a screen-positive result for their baby, this can be mitigated through effective communication. In a 2017 UK study, parents who received a screen-positive result and HCPs involved in communicating screening results both acknowledged the long-term impact of the initial phone call to deliver the screening results and agreed that there needed to be greater consideration of the optimal means of engaging with parents to communicate positive NBS results [8].

This evaluation aimed to engage with parents and HCPs in Ireland to better understand their experience of communication of screen-positive results for metabolic conditions through the NNBSP. Findings will add to the limited body of international literature on this important topic and will inform service improvement aimed at optimising communication of screening results in Ireland.

## 2. Materials and Methods

This was a service evaluation focused on the model for communication of a screen-positive result only, not confirmatory or diagnostic test results, i.e., at the time the result is communicated to the parent, the baby has had a screen-positive result for a metabolic condition, indicating increased risk of the condition, with further diagnostic testing required to determine whether the baby has the condition (screening result was a “true positive”; TP) or not (screening result was a “false positive”; FP). The approach was informed by the Donabedian [9] model for quality of care, comprising structure, process, and outcome measures.

The evaluation required engagement with two parent cohorts (a TP cohort and an FP cohort) and one HCP cohort.

### 2.1. Parent Engagement

#### 2.1.1. Population

The parent engagement element of the evaluation was designed in collaboration with the staff of the NCIMD. The TP population comprised parents whose children underwent screening in 2019–2023 inclusive and who received a positive initial screening result for metabolic conditions where subsequent diagnostic testing confirmed the diagnosis, i.e., the initial screening result was a true positive. A total of 121 babies with metabolic conditions were detected through the NNBSP in the period from 2019–2023; 34 were excluded following the application of exclusion criteria, yielding a total of 87 eligible for inclusion (see Table 1).

The FP population comprised parents whose children underwent screening in 2023–2024 inclusive, and who received a positive initial screening result for a metabolic condition, where subsequent diagnostic testing found the screening result to have been a false positive. A total of 117 babies received a false positive screening result for a metabolic condition through the NNBSP in the period from 2023–2024; 37 were excluded following the application of exclusion criteria, yielding a total of 80 eligible for inclusion (see Table 1).

Screening time periods were chosen to ensure adequate sample sizes, to ensure all metabolic conditions screened for were represented (screening for MCADD and GA-1 was introduced in 2018), and because the time periods were deemed sufficiently recent that parents would recall the experience of receiving an initial screen-positive result while mitigating the potential to cause additional distress in parents with more recent experience.

#### 2.1.2. Instrument

The evaluation instrument was a survey with minor variations between the TP and FP versions, the content of which was informed by the scope of the evaluation, the findings of the literature review, and expert opinion. The survey focused on parent experience of communication of NBS results, with questions on overall acceptability of NBS and attitudes towards programme expansion also included. Key information collected included: nature, quality, and quantity of information received at time of initial communication of screen-positive result; preference for information type and format; aspects of the communication that were particularly positive or negative; feelings at the time of communication of the result; and suggestions to improve the programme. Demographic data were collected to enable stratification of findings by key variables. Principles of data minimisation were followed. Pilots of the TP and FP surveys were undertaken with parents outside of the evaluation population (year of screening outside of inclusion criteria) to test and refine the evaluation instruments.

#### 2.1.3. Data Collection

TP data were collected over an approximate 6-week period in April and May 2025. Contact was attempted with all eligible parents. TP parents were contacted by staff at the NCIMD by phone, email, or both (depending on contact information on record in clinical service and whether a response was obtained at first attempt) to invite them to participate.

Parents wereinvited to complete the survey (i) via an electronic survey link emailed to them, or (ii) over the phone, or (iii) in person in clinic. The option of having the survey administered over the phone or in person (in an outpatient setting) by a member of the NCIMD who could provide support throughout was offered to TP parents due to the sensitive nature of the subject matter, and because these were parents of children with confirmed metabolic diagnoses and were well known to the metabolic services. Staff at the NCIMD had capacity to support this approach as parents were well known to them and it was anticipated that most parents would complete the survey via electronic link. This was indeed the case, with the majority of parents (*n* = 31/43) completing the survey via electronic link, 10 surveys completed over the phone, and 2 surveys completed by parents in clinic with a member of the metabolic team. Survey data were collected electronically through SmartSurvey.

FP data were collected over an approximate 3-month period from July to October 2025. Contact was attempted with all eligible parents. FP parents were contacted by phone by a CLN from the NNBSL and invited to complete the survey over the phone (interviewer-administered). Mother’s phone number is the only contact information routinely collected by the CLNs, so this was the method used to invite participation. The option of emailing a survey link was not offered, as it was felt that completion over the phone with the support of the CLN would maximise response rates in this cohort and allow for real-time clarification of parent queries around the questions being asked. In-person completion of the survey was not offered due to lack of capacity/resourcing to offer this option to the FP parent cohort. Survey data were collected electronically through SmartSurvey.

#### 2.1.4. Data Analysis

Data were analysed using JASP statistics program (version 0.19.3). Descriptive analysis was undertaken, as well as Chi-Square and Fisher’s Exact tests, to explore relationships between categorical variables. Due to small numbers, several predictor variables and some response options for outcome variables were re-categorised to allow meaningful comparisons. Inferential analysis could not be undertaken on a number of outcome variables due to high homogeneity of responses. Open-text responses were thematically analysed by one data coder. Themes were derived from the data. Themes were attached to data within the SmartSurvey platform. Illustrative quotes were selected to represent and reflect key themes identified.

### 2.2. HCP Engagement

#### 2.2.1. Population

The evaluation population was HCPs (pediatricians, neonatologists, nursing and midwifery staff, DLPs and other clinical staff) currently or previously working in one of the 19 public maternity hospitals/units in Ireland whose role includes communication of screen-positive results to parents. HCPs did not have to have previous experience of communicating NBS results in order to participate. It was not possible to determine the population size.

HCPs were asked about experience of communicating screening results for any/all conditions screened for under the NNBSP to support an understanding of whether confidence in delivering NBS results for non-metabolic conditions (CHT and CF), which are more common in the Irish population, differed to metabolic conditions.

#### 2.2.2. Instrument

The evaluation instrument was a survey, the content of which was informed by the scope of the evaluation, the findings of the literature review, and expert opinion. While the survey focused on HCP experience of communication of NBS results, additional questions on training and awareness of NNBSP and the appropriate role/grade of HCP to communicate screening results were also included. Key information collected included: awareness of the conditions included in the NNBSP and resources available to HCPs; experience of delivering screen-positive NBS results; confidence in delivering results; and views on the appropriateness of delivery of screen-positive NBS results in their current role. Principles of data minimisation were followed and, accordingly, only key demographic data were collected to enable stratification of findings by these variables. The survey was piloted internally within the Health Service Executive (HSE) Child Health Public Health team, with a primary focus on testing question routing.

#### 2.2.3. Data Collection

The survey information and survey link were disseminated through the lead Consultant Pediatricians and Neonatologists in the 19 maternity hospitals/units, as well as the Faculty of Pediatrics in the Royal College of Physicians in Ireland. An initial informational email with the survey email was issued, as well as a reminder email. The information email requested that the survey link be disseminated to any clinical colleagues with a role in communicating screen-positive NBS results to parents, acknowledging that practice may vary locally. Data were collected from September to October 2025.

#### 2.2.4. Data Analysis

Data were analysed as described in Section 2.1.4 above.

### 2.3. Ethical Considerations

Research Ethics Committee review was not required as this project was routine service evaluation [10] completed as Public Health Practice under Medical Officer of Health legislation including the Health (Duties of Officers) Order (1949), which sets out the statutory duties of Medical Officers of Health, including monitoring and protecting public health. However, ethics were a key consideration for the project group, and ethical principles [11] were followed at all times.

## 3. Results

### 3.1. Response Rates and Demographics

The response rates for the TP and FP parent cohorts were 49% (43/87) and 64% (51/80), respectively (see Table 2).

Demographics of parent cohorts are presented in Table 3. Respondents were primarily mothers, with some representation from fathers in the TP cohort (85% of respondents were mothers in TP cohort and 100% in FP cohort, *p* = 0.007). The ethnic profile of respondents broadly aligned with that of the overall population, and the majority were White Irish (74% in TP cohort and 80% in FP cohort, *p* = 0.58). In terms of area-level deprivation, respondents broadly aligned with the deprivation profile of children with inherited metabolic conditions [12], with no significant difference between TP and FP cohorts (*p* = 0.55). Both cohorts included representation from all six HSE regions.

The most commonly detected/suspected condition was PKU in the TP cohort (70%, including *n* = 2 hyperphenylalaninemia) and HCU in the FP cohort (39%). There was a significant difference between TP and FP cohorts in terms of the condition suspected (*p* < 0.001), reflecting variation in the performance and positive predictive value (PPV) of screening tests for individual conditions. At the time of the survey, 19% of the TP cohort had a family history of the condition, while none of the FP cohort had a family history (*p* = 0.001).

There was no denominator for the HCP survey, as it is not possible to determine all HCPs who are/have been tasked with delivering screen-positive NBS results. A total of 62 HCPs completed the survey, comprising a mix of clinical roles. The majority (64%) were in senior medical roles (registrar/consultant) and 21% were in senior nursing/midwifery roles. Almost all respondents (98%) were currently working in obstetric/neonatal/pediatric care, and the majority (74%) had more than 6 years’ experience delivering obstetric/neonatal/pediatric care (see Table 4).

### 3.2. Structure

Under the Donabedian model for evaluating the quality of healthcare, “structure” refers to all the factors that affect the context in which care is delivered, including the physical facility, equipment, and human resources, as well as organisational characteristics such as staff training [9].

#### 3.2.1. Role/Grade of HCP Delivering Screen-Positive Results

Under the current model [1], the DLP/pediatric registrar in the maternity hospital/unit of birth contacts the parent(s)/legal guardian(s) to communicate the screen-positive result. Our evaluation demonstrated heterogeneity in the role/grade of HCP tasked with delivering screen-positive results. Of the 62 HCPs who completed the survey, approximately two-thirds (*n* = 42, 68%) had previous experience of communicating a screen-positive NBS result to parent(s). The breakdown of these respondents, by professional role/grade, is illustrated in Figure 1. While the majority (*n* = 32, 76%) were senior medical and nursing (*n* = 6, 14%) grades, screening results had also been communicated to parents by more junior medical staff, including senior house officers (SHOs) (*n* = 2, 5%).

Among respondents to the parent surveys, 53% of those with TP results and 83% of those with FP results reported that the screening result was communicated to them by a doctor. Others reported being told by a range of other HCPs including a midwife (19% in TP cohort and 10% in FP cohort), nurse (16% in TP cohort and 2% in FP cohort), or another HCP (12% in TP cohort and 5% in FP cohort).

#### 3.2.2. Experience and Confidence in Delivering Screen-Positive Results

As described above, of the 62 HCPs who completed the survey, approximately two-thirds (68%) had previous experience communicating a screen-positive NBS result to parent(s), with CHT (69%, 27/39), PKU (36%, 14/39), and CF (31%, 12/39), the conditions which HCPs had most commonly communicated results for. The number of times they had done so ranged from 1–2 times to more than 10 times. Of those who had communicated a screen-positive result, 59% reported that they felt confident to tell the parent about the result. There was a significant difference in whether the HCP felt confident based on role/profession/grade, with consultant pediatricians demonstrating the greatest level of confidence (*p* = 0.046).

All respondents, including those who had never communicated a screen-positive result to a parent, were asked how confident they would feel communicating a screen-positive result to a parent today or in the future. Approximately two-thirds (68%) reported that they would be confident to do so. There was a significant difference in whether the HCP felt confident based on role/profession/grade (*p* = 0.03), with consultant paediatricians demonstrating the greatest level of confidence followed by consultant neonatologists. Respondents reported the highest level of confidence in relation to communicating a screen-positive result for more common conditions, including non-metabolic conditions such as CHT and CF, while lower levels of confidence were reported in relation to communicating a screen-positive result for rarer conditions such as MCADD and GA-1 (*p* = 0.006).

#### 3.2.3. Resources/Supports/Training

Of the 62 HCPs who completed the survey, just over half (52%) reported that they had received training/education in relation to the NNBSP. Those working in nursing or midwifery were significantly more likely to have received training/education (*p* < 0.001).

The majority of HCPs (85%, 53/62) correctly reported that between 8 and 12 conditions are screened for as part of the NNBSP. One-tenth (10%, 6/62) incorrectly reported that less than 8 conditions are screened for, and a further 5% (3/62) incorrectly reported that between 12 and 24 conditions are screened for. The majority of HCPs (82%, 51/62) reported that they were aware of all nine conditions screened for at the time of the evaluation, and the remaining 18% (11/62) reported that they were aware of some of the conditions screened for. The likelihood of being aware of all conditions did not differ significantly by role/profession/grade (*p* = 0.051) or years experience delivering obstetric/neonatal/pediatric care (*p* = 0.16).

Awareness of key information resources ranged from 52% to 95% (see Table 5). The most commonly used resources were the HSE website and the NNBSL. More than half of respondents (56%, 35/62) were aware of who the DLP was in their maternity hospital/unit.

Of those who had communicated a screen-positive result, 95% sought out additional information prior to contacting the parent. The most commonly used information sources were Google (59%), speaking to a more experienced HCP (51%), and the NNBSL (51%). The majority (78%) of those who sought additional information reported that they found all the information they needed. Sixty-seven percent of those who had communicated screen-positive results reported that they signposted the parent(s) to further information. The information sources most signposted were the HSE website and the NNBSL.

When presented with a list of resources and asked which would be useful to support delivery of NBS results to parents, all HCPs (100%, *n* = 56) reported that it would be useful to have an information resource that they could provide to parents with details of the relevant condition, implications of the screen-positive newborn bloodspot result, and next steps. Other resources which were perceived as being useful are detailed in Table 6.

#### 3.2.4. Acceptability of Current Model to HCPs

The majority (61%) of HCPs felt that delivering screen-positive NBS results to parents is an appropriate part of their role. HCPs were invited to comment on who/what grade of HCP or other staff member they felt should have the role of delivering screen-positive NBS results to parents. There were 21 responses to this question. Key themes identified included the opinion that delivery of NBS results should be medically led (indicated by 11 respondents), with specific suggestions including a Clinical Nurse Specialist or other specialist (*n =* 4), a member of the metabolic team (*n* = 3), a member of the team providing long term care to the baby (*n* = 2), a member of the team in the NNBSL (*n* = 2), and a “consultant or senior registrar trained in it” (*n* = 1). A selection of comments from HCPs in relation to the acceptability of the current model and the appropriate role to communicate screen-positive results is presented in Table 7.

### 3.3. Process

Under the Donabedian [9] model, “process” is the sum of all actions that make up healthcare, including diagnosis, treatment, preventive care, communication of test results, patient education, and actions taken by the patients or their families. Processes can be technical, i.e., pertaining to how care is delivered, or interpersonal, i.e., pertaining to the manner in which care is delivered.

While the current model does not specify the method for contacting parents regarding screen-positive results, established practice is for the information to be communicated over the phone, unless the baby is already being cared for as an in-patient. The majority (90%) of parents who ultimately received a TP result and 96% of those who ultimately received a FP result reported that they were notified of a screen-positive result over the phone.

The majority of parents reported that they were with a trusted person (friend/family member) at the time they received the screening result, but approximately one-third (28% in the TP and 39% in the FP cohort) were alone.

Further information on parent perception of the manner in which screening results were communicated is provided below.

### 3.4. Outcomes

Under the Donabedian [9] model, “outcomes” encompasses all the effects of healthcare on patients or populations, including changes to health status, behaviour, or knowledge, as well as patient and HCP satisfaction (Section 3.2).

#### 3.4.1. Quality and Quantity of Information Received

The evaluation found that, overall, approximately one-quarter (24%) of parents were happy with the amount of information provided at the initial communication of a screen-positive result (21% in TP cohort and 27% in FP cohort, *p* = 0.48), and 77% would have liked more information (77% in TP cohort and 71% in FP cohort, *p* = 0.76) (see Table 8). Approximately half of all parents (52%) felt that the information provided was “clear and easy to understand” (33% in TP cohort and 69% in FP cohort, *p* = <0.001).

The majority of parents reported being told the name of the condition suspected (67% TP, 65% FP, *p* = 1.00) and information on next steps (65% TP, 90% FP; *p* = 0.009) (Table 9). However, approximately one-third reported not being given this very basic information. Smaller proportions reported receiving basic information about the condition (16% TP, 20% FP, *p* = 0.61), being informed that their child might not have the condition (26% TP, 39% FP, *p* = 0.37), being reassured that the condition could be managed and that their child could live a healthy life (16% TP, 25% FP, *p* = 0.79), or being advised where to seek further information (2% TP, 2% FP, *p* = 1.00) (see Table 9).

Likelihood of reporting having received key information did not vary by demographic variables, such as ethnicity, deprivation, or time since screening. In the FP cohort, parents were significantly more likely to have been told that their child might not have the condition when the results were communicated by a nurse or midwife compared to a doctor (*p* = 0.04).

When asked what information they would like to have received, the majority of respondents (88.2–97.6%) across both parent cohorts said they would have liked to receive each of the information items listed in Table 10. In both TP and FP cohorts, parents reported a preference for information to be made available in multiple formats and on more than one occasion, e.g., verbally from an HCP, followed by written or electronic communication.

#### 3.4.2. Positive and Negative Aspects of How the Screen-Positive Result Was Communicated

Forty percent (17/43) of TP parents and 43% (22/51) of FP parents provided a response when asked whether there were any positive aspects of how they were told the screen-positive NBS result. The proportion of parents reporting a positive aspect did not differ significantly between cohorts (*p* = 0.834). Key themes identified included the kindness of the caller, reassurance provided, being afforded sufficient time on the phone call, being provided with clear information and useful advice, and having questions answered (see Table 11).

Seventy percent (30/43) of TP parents and 71% (36/51) of FP parents provided a response when asked whether there were any negative aspects of how the screening result was communicated to them. The proportion of parents reporting a negative aspect did not differ significantly between cohorts (*p* = 0.209). Key themes identified across both parent cohorts included insufficient information provided, and communication of a screen-positive result by HCPs who lacked the knowledge required to address parental questions and concerns. In addition, TP parents cited difficulty understanding or taking in the information and feeling no emotional attachment or support from the caller, and FP parents cited insufficient reassurance, feeling that the caller was rushed, not being afforded the opportunity to ask questions, not being told that the child may not have the condition, the caller causing an unnecessary level of panic, and not being directed to more information (see Table 11).

#### 3.4.3. Emotional Impact of Receiving a Screen-Positive Result

Parents described a range of emotions on receiving a positive NBS result for their baby, including shock, worry, and fear. Figure 2 illustrates the most cited responses from parents when asked to describe their feelings the first time they were told that “something had shown up in the heel prick test”, with larger font indicating more common responses. The emotional impact of the communication was relatively similar between both cohorts.

#### 3.4.4. Overall Experience and Attitudes Towards NBS

All parents (100%) in both the TP and FP cohorts reported they were glad their baby underwent screening (Figure 3). Support for expansion of the programme to screen for more conditions was high, at 95% in the TP cohort, and 84% in the FP cohort *(p* = 0.205) (Figure 3).

### 3.5. Suggestions for Improving the Current Model

While most HCPs (61%) felt that communication of screen-positive NBS results to parents was an appropriate part of their role, some suggested that this should be a more specialised role with results delivered by staff at the NNBSL, or by a consultant metabolic pediatrician. The majority of HCPs noted that additional supports would be useful to aid communication of screen-positive results to parents, including guidelines, expert contacts, condition-specific resources, and checklists (see Table 6).

When asked to provide suggestions as to how communication of NBS results could be improved, 86% and 82% of parents from the TP and FP cohorts, respectively, provided a response (*p* = 0.105). Key themes included provision of more information, delivery of information in a kind and reassuring manner ensuring that parents know that the suspected condition is manageable if ultimately diagnosed, delivery of the result by specially trained HCPs or those with increased education or training, signposting to additional information/provision of contact details of someone to call for further information, and early access to a peer support network of parents of children with metabolic conditions (cited by TP parents only) (see Table 12).

## 4. Discussion

This evaluation highlighted important contrasts between HCP and parent perspectives, with scope to significantly improve communication of screen-positive NBS results to parents in Ireland. Key considerations include the role/grade of HCP tasked with delivering screen-positive results to parents, the quality and quantity of information provided, and the method of communication.

Under the current Irish model, screen-positive results are communicated to parents primarily by senior medical and nursing staff, with variation in practice at local level. While this model ensures resilience and allows flexibility in line with local resources, the rarity of the conditions means that many of these clinicians will be asked to communicate screening results to parents very infrequently, limiting opportunities to become highly skilled in this complex activity. Communication of screen-positive NBS results requires not only accurate clinical knowledge but also the ability to assess and respond to parents’ differing informational and emotional needs. Parent and HCP suggestions for improvements to the Irish model included specialist-led communication, possibly via a dedicated liaison clinician or specialist team. Some suggested that initial contact should be made by staff of the metabolic services or NNBSL. While this approach should be considered, it would require appropriate resourcing to ensure resilience.

Additional options to improve communication under the current model would include routine liaison with a metabolic specialist before communicating screening results to parents, with 91% of HCPs in our evaluation reporting that this would be helpful. This aligns with international evidence demonstrating improved communication when clinicians consult with a metabolic pediatrician before and/or during disclosure [4], and with parent preferences and expectations; a 2022 systematic review found that parents preferred to receive initial results from HCPs knowledgeable about screening and the condition for which their infant screened positive, and who could communicate clearly and empathically [13]. Ideally, such individuals would be known, trusted providers, though being knowledgeable superseded familiarity [13].

Whatever model is in place, it is essential to ensure those delivering screening results receive appropriate training and support and have sufficient experience and expertise to maintain consistency and quality in communication. Of note, our evaluation demonstrated disparities in training opportunities, knowledge of the screening programme, and confidence in delivering screening results among HCPs. Approximately one-third of HCPs reported that they would not feel confident delivering a screen-positive result, and many expressed a desire for additional supports and resources, including access to specialist colleagues who they could consult prior to contacting parents, and checklists to guide this complex communication. Similar challenges are reported internationally. A survey of HCPs involved in communicating NBS results in the USA (including newborn screening coordinators, nurses, public health professionals, and a genetic counsellor) found that more than one-third of respondents had not received formal training in relation to the metabolic conditions screened for [14]. Gaps in HCPs’ knowledge of metabolic conditions screened were identified (mean rating of 3.5/5, where 1 is very limited and 5 is very good), as well as in relation to how to inform parents of abnormal results (mean rating 2.4/5) [14]. In England, despite HCPs investing a significant amount of time and resources to ensure good communication of screen-positive NBS results, there continues to be significant variation in practice, influenced by a lack of clear, definitive guidance, and a lack of resources [3].

Regarding the quality and quantity of information provided, just one-quarter of parents were happy with the amount of information received at the initial communication of a screen-positive result, and 77% would have liked more information. Additionally, only half of all parents felt that the information provided was “clear and easy to understand”. This contrasts starkly with the findings of the HCP survey, the majority of whom reported feeling confident in delivering screen-positive results. A notable finding highlighting the disparity between the parent and HCP experience is the fact that 67% of HCPs who had communicated screen-positive results reported that they signposted the parents to further information (most commonly the HSE website and the NNBSL), but only 2% of parents reported being advised of where to seek further information. While these findings may be impacted by recall bias, the current model could be strengthened to ensure all parents consistently receive a standardized suite of key information.

Challenges highlighted by the Irish evaluation mirror those reported internationally. An Italian study by Bani et al. found that parents were largely dissatisfied with the quality and depth of information provided by HCPs at the time of communication of screen-positive NBS results, and with the manner of communication, citing failure to create a caring, empathic, and safe environment [15]. A Dutch study by Haitjema et al. found that a majority of parents (60%, 68/113) were overall unsatisfied with the first communication of abnormal PKU metabolic screening results, and 66% indicated that wrong or no information about PKU was provided [4].

When asked what information they would like to have received, the majority of respondents across both parent cohorts in our evaluation said they would have liked to be told basic information about the condition, the chance their baby has the condition, reassurance that the condition is manageable, advice on where to find more information, a summary of the “next steps”, and contact details for a staff member who could provide more information if required. There were no significant differences between TP and FP cohorts, indicating that information needs at the initial communication of a screen-positive result are consistent, regardless of the eventual diagnostic outcome. Provision of this key information to all parents could be supported through the development of standardized resources to guide HCPs in delivering the initial communication, development of appropriate parent information leaflets to support a better understanding of what an initial screen-positive result means, and consideration of the need for follow-up electronic communication after the initial phone call.

While telephone communication of an initial screen-positive NBS result is appropriate given the clinical urgency and need for immediate action by the parent (i.e., bring the baby to hospital same day/next day for clinical evaluation and diagnostic testing), it has obvious limitations. These include limited capacity for non-verbal communication, limited time for parents to assimilate information and ask questions, and limited capacity to ensure optimal conditions for the parent to receive this communication, e.g., ensuring the parent is not busy at the time and is supported by a friend or family member if appropriate; our evaluation demonstrated that approximately one in three parents were alone at the time they received the screening result. Suggestions from parents surveyed in our evaluation included delivery of NBS results in person rather than over the phone. This aligns with the findings of a 2024 international scoping review of the psychosocial impact of false positive NBS results, which found that parents and HCPs often report a preference for face-to-face communication [16]. The reasons for this are complex and include providing a more personable approach, ability to adapt information based on the emotional response of the recipient, and concerns relating to the circumstances in which a parent is receiving a phone call, e.g., where they are and who they are with at the time, as being in a controlled environment with the support of a trusted person may result in a better experience [16]. While in-person communication of an initial screen-positive NBS result is unlikely to be feasible or clinically appropriate, HCPs delivering screening results should be supported to ensure the phone call is executed as effectively as possible.

Some of the limitations of phone communication could be mitigated by follow-up electronic communication. In our evaluation, parents reported a preference for information to be made available in multiple formats and on more than one occasion, e.g., verbally over the phone, followed by written or electronic communication. As noted by one parent; *“A follow-up text or email after the call would be useful because it’s hard to take in information over the phone. Once you hear there might be something wrong with your baby, it’s hard to focus on any other information or stats.”* This aligns with international evidence that parental distress limits information retention immediately after disclosure [17]. Our evaluation found that learning of a positive screening result engenders feelings of shock, fear, worry, and anxiety. The emotions experienced by parents in Ireland align with those described in the international literature. An Italian study published in 2024 found that approximately 78% (197/254) of parents reported a clinical level of distress and anxiety immediately following communication of a screen-positive NBS result, with one-third reporting post-traumatic symptoms and more than half reporting a “need for help” (as measured by an emotional thermometer) [6]. In line with other research [13], the study authors emphasised the importance of sensitive, jargon-free communication delivered with awareness of parents’ emotional state and adequate psychological support at this initial stage, regardless of the ultimate diagnostic outcome [6].

Importantly, despite the challenges in effective communication of screen-positive results, our evaluation demonstrated high parental support for NBS in Ireland, with 100% of parents across both TP and FP cohorts glad that their baby underwent screening and the majority in favour of programme expansion to screen for more conditions. Optimizing communication of screen-positive results would help to maintain the high level of parental trust in and acceptance of newborn bloodspot screening in Ireland.

### 4.1. Strengths and Limitations

Key strengths of this evaluation include multidisciplinary input to its design and delivery, with collaboration across programme, laboratory, and clinical staff involved in the delivery of the NNBSP and provision of care for screen-positive babies. Survey development was informed by international literature and expert input, and piloting and refinement of parent surveys was undertaken to ensure they were fit for purpose. The evaluation considers both parent and HCP perspectives, which enhances the credibility and validity of the results [18]. Given the relatively small size of the sample populations for the parent cohorts, contact was attempted with all eligible parents to invite them to participate, limiting the potential for sampling error and selection bias. Parent response rates were high compared with international studies [5,15], likely reflecting recruitment through trusted clinical teams. The HCP sample invited representation from all 19 maternity units, and the sample included a wide range of disciplines and grades.

Limitations of the evaluation include those inherent to the study of rare diseases, with limited study populations from which to recruit participants and reliance on retrospective self-reporting with potential for response and recall bias. However, the international literature suggests that recall bias is less likely for highly salient events [19]. For parent cohorts, knowledge of the diagnostic outcome may have influenced recollection and attitudes towards the initial communication of a screen-positive result. For some TP parents, experiences may have been impacted by the COVID-19 pandemic. The majority of parent responses were from mothers and cannot be considered representative of the experience of fathers of screen-positive babies. This arose due to the fact that “mother’s phone number/email address” was the most commonly held contact information by the relevant clinical services. Findings of the HCP survey cannot be considered representative.

### 4.2. Conclusions

This evaluation represents the first formal evaluation of the model for communication of screen-positive NBS results in Ireland, providing valuable insights to inform programme delivery, expansion, and quality improvement. Important contrasts between the parent and HCP experience are noted. While the current model is broadly acceptable to HCPs and the majority feel confident to deliver screen-positive results, important gaps remain in confidence, training, and support, and the parent experience is inconsistent and often suboptimal. These challenges mirror those reported internationally. Both parents and HCPs offered valuable recommendations for how communication of screen-positive results can be improved, including through greater standardisation, enhanced specialist support, and improved parent and HCP resources. These recommendations will inform ongoing service improvement to further strengthen this critical service for the Irish population.

## Figures and Tables

**Figure 1:**
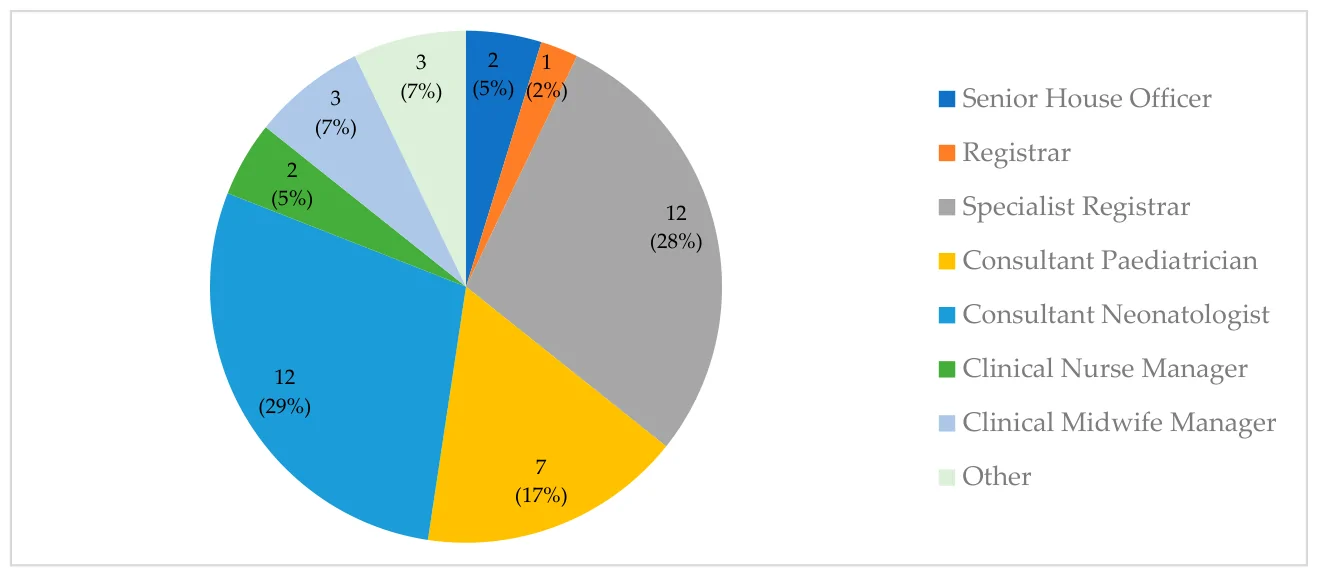
Respondents who had communicated screen-positive result by professional role/grade (*n* = 42).

**Figure 2:**
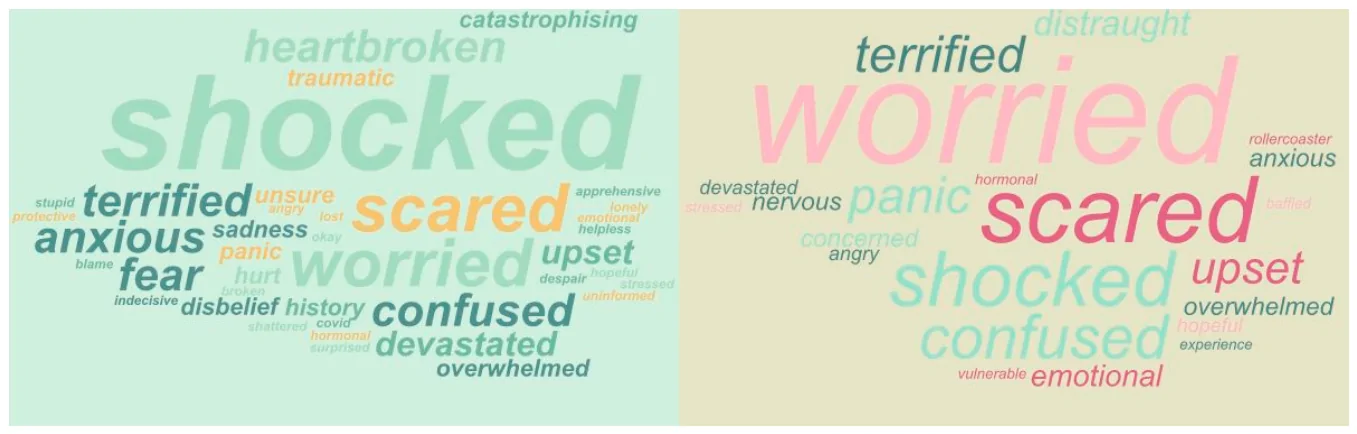
Parental feelings on being told about their baby’s positive NBS result (TP (**left**) and FP (**right**) cohorts).

**Figure 3:**
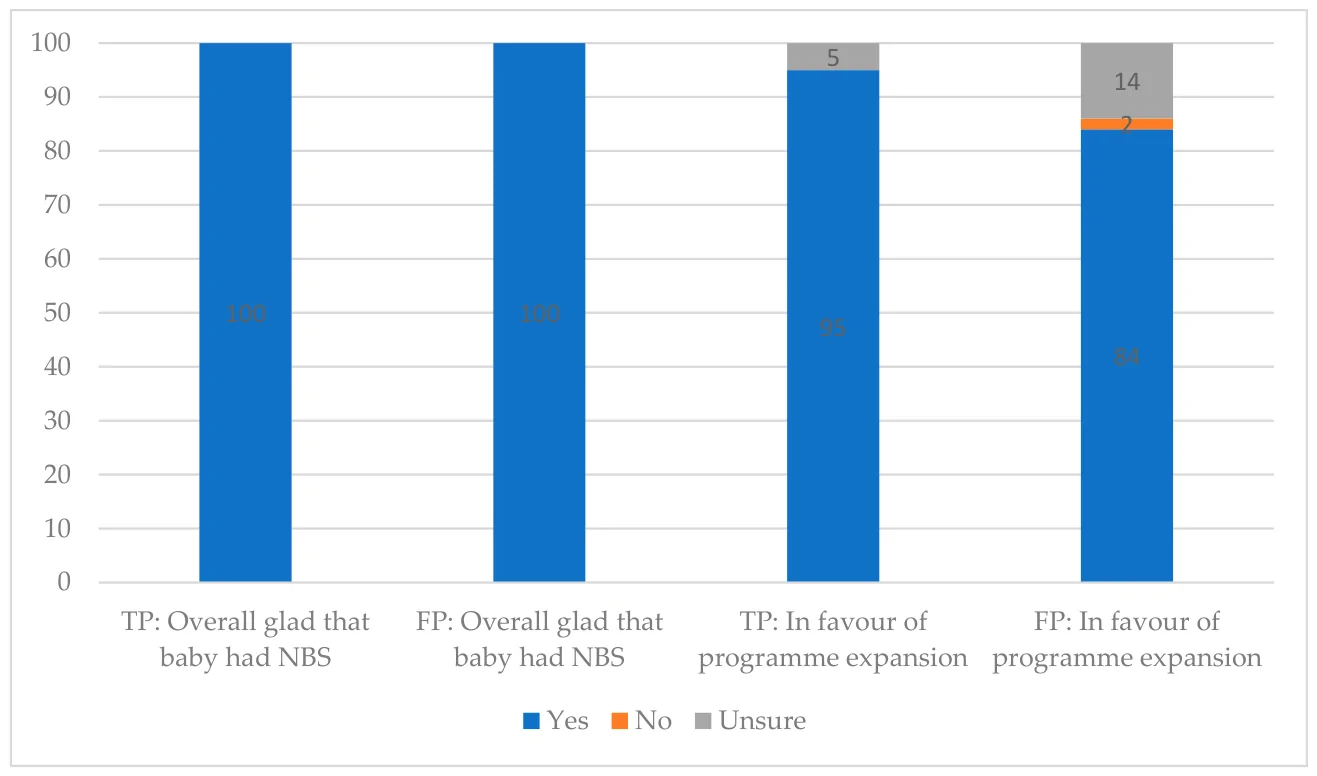
Parent attitudes towards NBS and expansion of NNBSP (%).

**Table 1:** Overview of study population and exclusion criteria.

	TP	FP
Condition		
PKU	71 (including hyperphenylalaninemia, *n* = 17)	1
MSUD	0	15
HCU	6	48
CGal	24	22
MCADD	18	16
GA-1	2	15
Total	121	117
Exclusion criteria		
Baby was the second or subsequent child diagnosed with the condition in the same family	26	-
Baby preterm in NICU *	-	18
Baby unwell in hospital at time of screening	-	8
Baby diagnosed with a different metabolic condition	-	6
NCIMD clinical judgement regarding appropriateness for inclusion in context of challenging clinical circumstances	2	3
Second twin	-	2
Known antenatal diagnosis from non-invasive prenatal testing	1	-
Lost to follow-up e.g., moved to another jurisdiction	5	-
Total sample population	87	80

* NICU = Neonatal intensive care unit.

**Table 2:** Response rates by condition in parent cohorts.

	TP		FP	
	*n*	%	*n*	%
Response rate				
Overall	43/87	49.4%	51/80	63.8%
PKU (including hyperphenylalaninemia, *n* = 2)	30/56	33.3%	-	-
CGal	5/15	53.6%	8/13	61.5%
MCADD	5/9	55.6%	8/11	72.7%
HCU	2/5	40.0%	20/33	60.6%
GA-1	1/2	50.0%	5/10	50.0%
MSUD	-	-	10/13	76.9%

**Table 3:** Demographics of parent cohorts.

	TP		FP		Comparison
	*n*	%	*n*	%	*p*-Value
Survey completed by					0.007
Mother	36/43	83.7%	51/51	100%	
Father	6/43	14.0%	0/51	0%	
Mother and father	1/43	2.3%	0/51	0%	
Ethnicity					0.58
White Irish	32/43	74.4%	41/51	80.4%	
White Irish Traveller	5/43	11.6%	0/51	0%	
Any other White background	6/43	14.0%	6/51	11.8%	
Black or Black Irish	0/43	0%	0/51	0%	
Asian or Asian Irish	0/43	0%	4/51	7.8%	
Area level deprivation ^1^					0.55
Very disadvantaged or disadvantaged	3/37	8.1%	7/51	13.7%	
Marginally above or below average	23/37	62.2%	33/51	64.7%	
Affluent or very affluent	11/37	29.7%	11/51	21.6%	
Condition suspected/diagnosed					<0.001
PKU	28/43	65.1%	-	-	
CGal	5/43	11.6%	8/51	15.7%	
MCADD	5/43	11.6%	8/51	15.7%	
HCU	2/43	4.7%	20/51	39.2%	
Mild PKU/hyperphenylalaninemia	2/43	4.7%	0/51	0%	
GA-1	1/43	2.3%	5/51	9.8%	
MSUD	-	-	10/51	19.6%	
Family history of the condition					0.001
None	35/43	81.4%	51/51	100%	
Parent has another child with this condition	3/43	7.0%	0/51	0%	
Another family member has this condition	5/43	11.6%	0/51	0%	

^1^ Based on the Pobal HP Deprivation Indices. This index shows the level of overall affluence and deprivation of areas and is updated for each national census.

**Table 4:** Key demographics of HCP respondents (*N* = 62).

	*n*	%
Working in obstetric/neonatal/pediatric care		
Currently working in obstetric/neonatal/pediatric care	61	98%
Previously worked in obstetric/neonatal/pediatric care	1	2%
Role/profession/grade		
Senior House Officer (SHO)	5	8%
Registrar	1	2%
Specialist Registrar	14	23%
Consultant Pediatrician	11	18%
Consultant Neonatologist	13	21%
Staff nurse	1	2%
Clinical Nurse Manager	4	6%
Clinical Midwife Manager	9	15%
Other	4	6%
Experience in delivering obstetric/neonatal/pediatric care		
Less than 1 year	1	2%
1–3 years	5	8%
3–6 years	10	16%
More than 6 years	46	74%

**Table 5:** Awareness of screening resources among HCPs (*n* = 62).

	Aware of and Have Used This Resource *n* (%)	Aware of but Have Not Used This Resource *n* (%)	Not Aware of This Resource *n* (%)
Practical Guide to Newborn Screening	28 (45%)	13 (21%)	21 (34%)
NNBSL	44 (71%)	9 (15%)	9 (15%)
HSE website	46 (74%)	13 (21%)	3 (5%)
Metabolic.ie	16 (26%)	16 (26%)	30 (48%)
Children’s Health Ireland website	27 (44%)	22 (35%)	13 (21%)

**Table 6:** Reported usefulness of resources by HCPs (*n* = 56).

	Would Be Useful *n* (%)	Would Not Be Useful *n* (%)	Unsure Whether This Would Be Useful *n* (%)
Information resource that you can provide to parent(s) with details of the relevant condition, implications of the screen-positive newborn bloodspot result and next steps	56 (100%)	0 (0%)	0 (0%)
Information for HCPs with details of the relevant condition, implications of the screen-positive result and next steps	55 (98%)	0 (0%)	1 (2%)
Contact details for an informed expert who HCPs can direct the parents to if they want further information, e.g., a staff member in the NNBSL, or a member of the metabolic team if a metabolic condition is suspected	53 (95%)	1 (2%)	2 (4%)
Contact details for a specialist that HCPs can talk to before delivering the result to parents e.g., contact details for Consultant Metabolic Pediatrician if metabolic condition is suspected	51 (91%)	1 (2%)	4 (7%)
A checklist to guide the conversation between the HCP and parent regarding the screen-positive result	48 (86%)	2 (4%)	6 (11%)
Communication/sensitivity training	40 (71%)	7 (13%)	9 (16%)
Regular continuing professional development on NBS	39 (70%)	10 (18%)	7 (13%)
Access to a brief video resource on how to communicate screen-positive results to parents	36 (68%)	13 (23%)	5 (9%)
A competency framework for HCPs delivering screen-positive results	37 (66%)	11 (20%)	8 (14%)
eLearning module on delivering screening results to parents	32 (57%)	12 (21%)	12 (21%)
Regular clinical supervision and emotional support for all staff engaged in such work	27 (48%)	10 (18%)	19 (34%)

**Table 7:** Selection of HCP comments in relation to appropriate role to communicate screen-positive results *.

*“It is part of the role for anyone working with babies to be able to communicate with families re positive screening results.”*
*“All general pediatricians/neonatologists should have a basic understanding and ability to communicate information to parents on all the conditions tested for by the heel prick test. However, parents should have immediate access to a specialist who has extensive experience in the particular condition their child may have. Parents’ initial questions are often about the day-to-day practicality of what the condition will look like for their child and long-term prognosis. A specialist with lived clinical experience of the condition is best placed to discuss this.”*
*“The parents and family already have a relationship with our service so feel it is appropriate for us to tell them. However, it would be great to have more training on how to give information to families and also have access to written information to give to families. Would be beneficial to have option to have these leaflets in other languages too.”*
*“I think this information should predominantly come from the team who will treat and follow the condition.”*
*“I think a person with the appropriate specialised knowledge of the condition in question, ideally from the metabolic team/National Newborn Bloodspot Screening Lab should be the person to impart this information to the parents from the very start so that if parents have very specific, detailed questions, they can get the answers they need straight away.”*
*“Every time I have felt like the ‘middle man’ I found it hard that it’s generally parents that you haven’t met either. I understand that it’s useful as a link for babies that need their bloods done e.g., CHT however for those that need to go straight to Temple Street I don’t think that it’s necessary for us to call them as the middle man that won’t see them, don’t know enough about the conditions to answer their immediate questions.”*

* Selected comments were chosen because they are illustrative of key themes identified.

**Table 8:** Parent satisfaction with information received.

	TP (*N* = 43)		FP (*N* = 51)		
	*n*	%	*n*	%	*p*-Value
Happy with amount of information provided	9/43	21%	14/51	27%	0.48
Would have liked more information	33/43	77%	36/51	71%	0.76
Information provided was clear and easy to understand	14/43	33%	35/51	69%	<0.001

**Table 9:** Information received by TP (*n* = 43) and FP (*n* = 51) parents.

	TP		FP		
	*n*	%	*n*	%	*p*-Value *
Name of the condition suspected					1.00
Told	29/43	67.4%	33/51	64.7%	
Not told	10/43	23.3%	11/51	21.6%	
Do not remember	4/43	9.3%	7/51	13.7%	
Basic information about the condition suspected					0.61
Told	7/43	16.3%	10/51	19.6%	
Not told	31/43	72.1%	34/51	66.7%	
Do not remember	5/43	11.6%	7/51	13.7%	
That their child might not have the condition					0.37
Told	11/43	25.6%	20/51	39.2%	
Not told	24/43	55.8%	28/51	54.9%	
Do not remember	8/43	18.6%	3/51	5.9%	
That the condition could be managed and their child could live a healthy life					0.79
Told	7/43	16.3%	13/51	25.5%	
Not told	30/43	69.8%	37/51	72.5%	
Do not remember	6/43	14.0%	1/51	2.0%	
Advice on where to find more information					1.00
Told	1/43	2.3%	1/51	2.0%	
Not told	36/43	83.7%	44/51	86.3%	
Do not remember	6/43	14.0%	6/51	11.8%	
Clear information on the “next steps”					0.009
Told	28/43	65.1%	46/51	90.2%	
Not told	14/43	32.6%	5/51	9.8%	
Do not remember	1/43	2.3%	0/51	0%	

* Do not know excluded from analysis.

**Table 10:** Preference for information in TP and FP parents.

Would Like to Have Been Told	TP		FP		
	*n*	%	*n*	%	*p*-Value
Basic information about the condition suspected	39/41	95.1%	48/51	94.1%	1.00
The chance that their baby has the condition	36/41	87.8%	48/51	94.1%	0.46
That the condition could be managed and their child could live a healthy life	40/41	97.6%	48/51	94.1%	1.00
Advice on where to find more information	37/41	90.2%	49/51	96.1%	0.40
Exactly where to bring their baby	40/41	97.6%	49/51	96.1%	1.00
Summary of the “next steps”	39/40	97.5%	49/51	96.1%	1.00
How quickly they need to take their baby to hospital	37/40	92.5%	49/51	96.1%	0.32
Contact details for a staff member who could give more information	40/41	97.6%	45/51	88.2%	0.13

**Table 11:** Selection of parent comnments in relation to positive and negative aspects of the initial communication *.

Positive Aspects
*“The nurse was very calm and nice over the phone, giving me all the instructions and answering my questions.”* (TP parent)
*“Lady we spoke to…was very calm and you could tell she knew how to deal with worried, crying people on the phone.”* (TP parent)
*“They were really nice on the phone, they used plain simple language to explain galactosemia because I had never heard of it. They were calm which made us less panicked.”* (FP parent)
*“The doctor was great. He was really kind and patient and explained everything really clearly and in simple language that we could understand.”* (FP parent)
Negative Aspects
*“The midwife on the phone had little to no knowledge of PKU. Although it will always be distressing to parents to receive a phone call of that nature, when you are being told to bring your child to hospital without delay, the caller should, at the very least, have sufficient knowledge of the condition to re-assure parents’ concerns.”* (TP parent)
*“It felt like a quick call and the person did not know much about the condition and little information about it was given. I think directed to a website and peers would have been invaluable.”* (TP parent)
*“The lack of explanation and information. We weren’t given a chance to ask any questions. The doctor didn’t even tell us what GA-1 was or that our baby might not have it we were basically just told to bring him to hospital.”* (FP parent)
*“The whole call was so unprofessional. To receive a call from a doctor telling you to bring your baby straight to hospital and then for him not to be able to explain why and what was wrong with your baby is just shocking. He just kept saying I have been told to ring you. I will never forget it as long as I live.”* (FP parent)

* Selected comments were chosen because they are illustrative of key themes identified.

**Table 12:** Selection of parent suggestions for improvements to the communication of NBS results *.

*“That initial phone call must be more than someone telling you to take your child to hospital. The caller must be able to explain the condition, what it entails and most importantly, re-assure the parents that the child will be just like every other child, albeit they will eat a little differently.”* (TP parent)
*“Yes more time provided on call, information about condition, directed to a website and network of peers.”* (TP parent)
*“A follow-up text or email after the call would be useful because it’s hard to take in information over the phone. Once you hear there might be something wrong with your baby, it’s hard to focus on any other information or stats.”* (FP parent)
*“Train the doctors making the calls to have the basic knowledge of what they are discussing on the phone with parents and to have communication skills. You simply should not be allowed to call new parents and tell them something is wrong with their newborn baby without being able to explain!”* (FP parent)
*“Instead of a phone call with the result, to be brought in face to face to get the results from a person who has some knowledge of the condition and can answer questions and offer support and kindness.”* (TP parent)

* Selected comments were chosen because they are illustrative of key themes identified.

## Data Availability

Data are unavailable due to privacy and ethical restrictions.

## Abbreviations

The following abbreviations are used in this manuscript:

CF	Cystic Fibrosis
CGal	classical galactosaemia
CHT	Congenital Hypothyroidism
CLN	Clinical Liaison Nurse
FP	False Positive
GA-1	glutaric aciduria type 1
HCP	Healthcare Professional
HSE	Health Service Executive
MCADD	medium chain acyl co-A dehydrogenase deficiency
NBS	Newborn Bloodspot Screening
NCIMD	National Centre for Inherited Metabolic Disorders
NICU	Neonatal Intensive Care Unit
NNBSL	National Newborn Bloodspot Screening Laboratory
NNBSP	National Newborn Bloodspot Screening Programme
PKU	phenylketonuria
SCID	severe combined immunodeficiency
SHO	Senior House Officer
SMA	Spinal Muscular Atrophy
TP	True Positive
